# Parents’ Experiences of Caring for Their Child at the Time of Discharge After Cardiac Surgery and During the Postdischarge Period: Qualitative Study Using an Online Forum

**DOI:** 10.2196/jmir.9104

**Published:** 2018-05-09

**Authors:** Jo Wray, Katherine Brown, Jenifer Tregay, Sonya Crowe, Rachel Knowles, Kate Bull, Faith Gibson

**Affiliations:** ^1^ Charles West Division Great Ormond Street Hospital for Children NHS Foundation Trust London United Kingdom; ^2^ Clinical Operational Research Unit University College London London United Kingdom; ^3^ MRC Centre of Epidemiology for Child Health UCL Institute of Child Health London United Kingdom; ^4^ Centre for Outcomes and Experience Research in Children's Health, Illness and Disability Great Ormond Street Hospital for Children NHS Foundation Trust London United Kingdom; ^5^ School of Health Sciences Faculty of Health and Medical Sciences University of Surrey Guildford United Kingdom

**Keywords:** congenital heart disease, parents, online forum, isolation

## Abstract

**Background:**

Congenital heart disease (CHD) is the most common class of birth defects, which encompasses a broad spectrum of severity ranging from relatively minor to extremely complex. Improvements in surgery and intensive care have resulted in an increasing number of infants with the most complex lesions surviving after surgery until the time of discharge from the hospital, but there remain concerns about out-of-hospital mortality, variability in how services are provided at the time of discharge and beyond, and difficulties experienced by some families in accessing care.

**Objective:**

As part of a mixed-methods program of research, this study aimed to elicit parental experiences of caring for a child with CHD after hospital discharge following a cardiac surgery and collect information to inform interviews for a subsequent stage of the project.

**Methods:**

A closed online discussion group was set up via the main Facebook page of the Children’s Heart Federation (CHF), a national charity offering support to children with heart disease and their families. The discussion group was advertised through the charity’s webpage, and interested participants were directed to the charity’s Facebook page from where they could access the closed Facebook group and respond to questions posted. The CHF moderated the forum, and the research team provided questions to be posted on the forum. Responses were collated into a single transcript and subjected to thematic analysis.

**Results:**

The forum was open for 4 months, and 91 participants (mean age 35 years, range 23-58 years, 89 females, 89 parents, and 2 grandparents) submitted demographic information and were given access to the closed forum group. A common experience of isolation emerged from the data, with descriptions of how that isolation was experienced (physical, social, knowledge) and its psychological impact, together with the factors that made it worse or better. Woven through this theme was the notion that parents developed expertise over time.

**Conclusions:**

The use of an online forum provided a means for eliciting data from a large number of parents regarding their experiences of caring for their child after hospital discharge following cardiac surgery. Parents engaged with the forum and were able to articulate what went well and what went less well, together with sharing their stories and supporting each other through doing so. Some parents clearly found participating in the forum a positive experience in itself, demonstrating the potential of social media as a mechanism for providing support and reducing isolation. Information gained from the forum was used to shape questions for interviews with parents in a subsequent phase of the study. Furthermore, the themes identified in the online forum have contributed to identifying ways of improving the provision of care and support for parents of high-risk babies following discharge after cardiac surgery.

## Introduction

**Figure 1:**
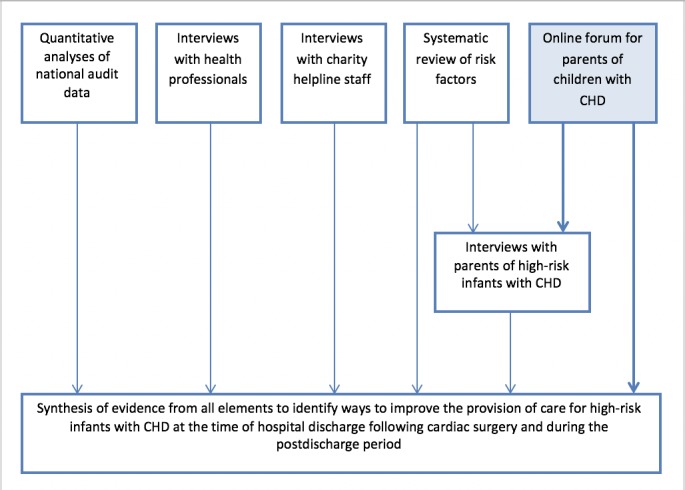
Elements of the research program. CHD: congenital heart disease.

Congenital heart disease (CHD) affects approximately 8 in every 1000 live births, and every year in the United Kingdom, approximately 2800 infants undergo heart surgery, with those at the more severe end of the disease spectrum often leaving hospital requiring ongoing care, such as tube feeding, multiple medications, and daily monitoring of oxygen saturations [1,2]. Improvements in surgery and intensive care have resulted in increasing numbers of infants with the most complex lesions surviving after surgery until the time of discharge from hospital but this has been accompanied by concerns about out-of-hospital mortality, variability in how services are provided at the time of discharge and beyond, and difficulties experienced by some families in accessing care. To understand and address these concerns, a multidisciplinary mixed-methods program of research was undertaken (Figure 1), including a systematic review [3], quantitative analyses of national audit data [4,5], and interviews with key stakeholders (parents, health professionals, and charity helpline staff) [6-8]. The overall aim was to synthesize the information gathered from each of the data sources to identify ways in which care could be improved for high-risk infants with complex cardiac conditions at the time of discharge and during the postdischarge period [9].

The focus of this paper is to present a discrete element of the study that centered on an online forum method used to address the following 2 aims:

1. Elicit parental experiences of caring for a child with CHD after discharge following cardiac surgery
2. Collect information from parents of children with CHD to inform the in-depth interviews (Figure 1)

A further aim emerged, which was to comment on the use of an online forum in the context of this study.

In this online forum part of our study, we particularly sought to explore the parents’ experiences of taking a baby home after congenital heart surgery. Elevated levels of distress, anxiety, and depression have been reported in parents of children who have undergone cardiac surgery, particularly those for whom their baby’s surgery was recent and those with more complex heart conditions [10,11]. Parents are often discharged with suboptimal levels of information about their child’s condition [12] and their children, particularly the most fragile infants, need skilled care once home after discharge [13], all of which can result in significant parental burden. A process of “safeguarding survival” has been described in parents of infants at highest risk of dying after discharge, whereby parents safeguard their child’s survival through taking charge of their infant’s care at home, protecting them from infection, and drawing on the support of others (extended family) to help with the care and monitoring of their child [14]. This has been developed further into a theory of “parenting under pressure,” which highlights the specific challenges parents face but also focuses on parental ability, resourcefulness, and resilience [15]. However, although such research provides important and valuable insights into parenting fragile, high-risk infants, the reliance on face-to-face interviews as the mechanism for data collection has excluded many parents from participating and also prevented us from accessing the insights from parents sharing experiences and opening up to each other.

Parents of children with significant health conditions can find it challenging to participate in face-to-face research for a variety of practical reasons; however, those who are difficult to reach via traditional data collection methods, such as fathers, those who are more geographically remote, and those from ethnically and culturally diverse cultural backgrounds often have important and salient contributions to make. Recent years have seen a significant increase in the use of online social networks, with evidence suggesting that approximately two-thirds of adults in the United States and Europe now use social media [16,17]. Social networks provide a quick and easy means of sharing ideas, information, and opinions; have a broad population reach; and are widely used in every sphere of life [16,18]. Facebook is the dominant social network worldwide and many health-related groups have arisen on Facebook that are predominantly used for raising awareness, social support, and fundraising. Developments in technology have also resulted in an increasing use of electronic methods to collect data for research, and there is evidence to support their feasibility, the prompt responses of participants, the richness of data collected by these means, and fewer human errors [19-21]. One method of electronic data collection is the online forum, which allows asynchronous interactions, whereby participants are able to join discussions at their own convenience, in contrast to methods that require synchronous interactions, such as chat groups. They have been reported to be relatively easy to use, safe, accessible, and observable, and it has also been suggested that they offer a more comfortable mechanism for the discussion of sensitive or personal health issues and are a feasible alternative to more traditional research tools, such as face-to-face focus groups [22-24]. They also provide participants with a “free rein” to express their views, such that responses may include valuable detail beyond that which was originally asked. Furthermore, they offer flexibility to researchers and participants alike, thus reducing participant burden and pressures of time.

## Methods

We used an Internet forum to elicit views on the information and support that parents were given at the time of discharge after their baby’s surgery and during the postdischarge period and their experiences of caring for their baby and interacting with health professionals in primary, secondary, and tertiary services.

### Data Collection

The online forum was facilitated via the Children’s Heart Federation (CHF) through its Facebook page. CHF is a national charity and is an umbrella organization representing approximately 12,000 children with heart disease and their families. They have an active presence and following on Facebook and Twitter, and at the time of setting up the online forum, the CHF Facebook page had around 3120 members. CHF has experience of running online forums, and as they are a national charity, their forums are accessed by parents of children treated at each of the specialist pediatric cardiac centers in the UK, thus providing us with the potential to include a more representative population of parents than we could achieve otherwise.

CHF entered into a contractual arrangement with us, and a fixed fee was agreed upon for their role in facilitating and managing the forum. A closed online discussion group was set up via their main Facebook page. The discussion group was advertised through the charity’s webpage, and interested participants were then directed to the charity Facebook page, where they could access more detailed information about the larger study and the online forum in particular. Information was provided about the rationale for the study, the role of the forum, how information from the forum would be used, and issues relating to confidentiality and anonymization of forum posts. The potential for touching on sensitive topics and that some bereaved families may contribute to the forum were acknowledged, and contact details for the CHF helpline were provided should participants want to talk to someone further about any issues that were raised on the forum. If people wanted to participate, they were required to provide some basic demographic information (their age, gender, ethnicity, and geographical region). Once this information was received by CHF, they were given access to a private or “closed” Facebook group and were able to begin responding to questions posted there. CHF was responsible for all day-to-day running and moderation of the forum in line with a standard operating procedure developed in collaboration with the research team, which included processes for managing inappropriate or offensive messaging and distressed users as well as procedures for running the forum. The research team provided questions to be posted on the forum at the start of the process, and CHF decided when new questions should be posted based on participant responses and the rate of responding. CHF was specifically asked to probe further if they noticed any of the following issues in participant responses:

1. Social and practical issues, for example, financial, educational, and transport issues
2. Issues to do with language or cultural differences
3. Difficulties accessing support in the community
4. Understanding information from health care providers

Forum responses were pseudonymized by CHF before being sent to the research team in a weekly update. CHF assigned participant numbers to individual respondents, but did not undertake any editing of responses before sending them to the research team.

Ethical approval was granted by the local National Research Ethics Service Committee London-Central (Reference: 12/LO/1398).

### Data Analysis

Responses were collated into a single transcript (78 pages), and thematic analysis was used to analyze forum responses according to recommendations made by Coffey and Atkinson [25]. A conceptual model was built and discussed with all members of the research team, showing clearly the relationships between the themes. The analysis proceeded as follows:

1. The transcript was analyzed as a whole. It was read, and notes were made in the margins on interesting or significant points said (JT, FG, and JW).
2. Codes were attached to segments of data. These segments could be one word, a phrase, or a sentence. The codes were a summary of what a parent seemed to be referring to or describing.
3. After the transcript had been coded, segments of data with similar codes were brought together to create categories containing data that shared a common element.
4. The categories were discussed, refined, and used to generate themes (JT, FG, and JW). As meanings can change when phrases are isolated, the original contexts of the phrases within the themes were checked.
5. Each theme was given a name that aimed to capture all the elements within that theme.
6. Agreement and understanding were gauged during discussions until consensus was reached; there were in fact few discrepancies. Working in this manner, we safeguarded against an interpretation representing the subjectivity of the observer more than the object of study.

## Results

### Study Participants and Overview of Responses

The forum ran for 4 months, during which time a total of 91 participants (mean age 35 years, range 23-58 years, 89 females, 89 parents, and 2 grandparents) submitted demographic information and were given access to the closed forum group. Participants came from all over the United Kingdom and were predominantly of white British ethnicity (85 of 91 participants). Of these, 73 parents participated in the forum discussion and most responded to between 1 and 5 questions. Neither of the 2 grandparents contributed to the forum discussion. Although data were not collected about the children themselves, it was evident from the responses that they had a wide range of cardiac diagnoses, including as part of genetic syndromes, and that they spanned a wide age range (infant to teenager), although most were of preschool age. A total of 19 questions were posted, although there was an overlap between some of the questions to probe for further detail and encourage other participants to respond. The first question had 43 responses and the final question had 39 responses, with the number of responses to individual questions ranging from 14 to 43. The questions with the most responses were those about information provided about caring for their baby at home (symptoms and how prepared they felt), community support (particularly experiences with general practitioners and health visitors [HVs]), and support from cardiac liaison nurses. Parents also responded to others’ posts, offering support and sharing experiences, and toward the end of the forum, parents became more open in identifying with other parents’ experiences.

Emerging from the data was an experience of isolation, as one parent said:

It’s a pretty lonely place.

**Figure 2:**
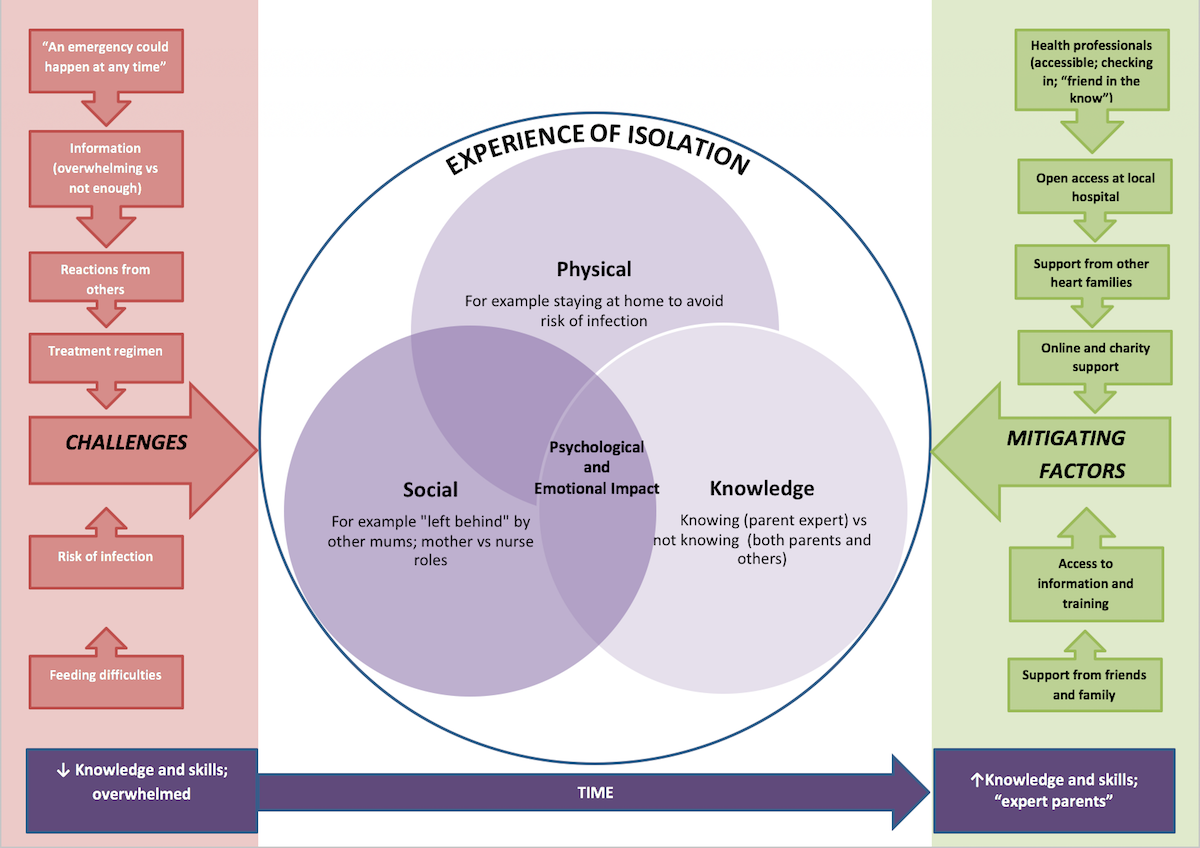
Parental experiences of caring for their child with congenital heart disease.

This was described in terms of the way in which that isolation was experienced (physical, social, knowledge) and the resulting psychological impact, together with the factors that made that worse or better (challenging and mitigating factors). Threaded through this theme was the notion of time, revealing parents developing expertise, where parents moved from feeling overwhelmed and lacking in knowledge and skills to becoming “expert parents” with a corresponding increase in their knowledge and skills (Figure 2). We present here these themes drawing on salient quotes that best illustrate our findings.

### Physical Isolation

A number of parents described being physically isolated as a result of having a baby with CHD:

[I] didn’t see a soul…from leaving hospital to returning for review.

The physical isolation was sometimes due to parents’ own anxieties and concerns, particularly about the risk of infection. Some parents felt that they could not go out to places where they would be in contact with other parents and babies:

I felt it too risky to go to clinic, with infections.

There were other parents who not only feared taking their baby out but also worried about visitors bringing infection into their home:

It was all such a huge shock, on discharge we were told she was like any other baby, but obviously there were differences. I was very scared about taking her out and catching a cold or something, or visitors bringing in germs.

In other situations, parents identified that professionals—particularly HVs—were anxious about mothers taking their babies to a busy clinic, preferring instead to make home visits, but this in turn could compound the challenges of isolation that the families were facing:

[My health visitor] was nervous about me going to baby clinics etc for weekly weights but I needed to get out.

Even when the visits themselves were perceived positively, there was still an isolating element to them:

...home visits were lovely but made me feel more isolated.

### Social Isolation

Parents described their feelings of social isolation in terms of interactions with other parents and about the support parents of a child with CHD received from support groups. Being unable to participate in “normal” mother and baby activities resulted in mothers feeling isolated from other new mothers, with the impact sometimes having consequences beyond the first weeks and months:

Loads of mum formed their friendships at that time. I was left behind and still am because of his additional needs.

Mothers described how they wanted to be like others, but in several instances, the particular needs of their baby singled them out from other parents, with a resulting isolating impact:

Went to mother and baby group once to get him weighed and never went again as was so upset by HV and other mums looking at your child's scar and not talking.

A number of parents were quite specific in their descriptions of what they considered to be failings in the support they were given and the isolating effect this had:

Absolutely no support groups...or help...feel quite isolated sometimes.

In contrast, other parents reported feeling well supported by their local community team:

Health visitor was good and did the best she could for us despite not having all the answers about concerns regarding baby’s heart defect. She came almost every week to weigh him …she contacted other professionals to seek advice or support for us. She was a welcome face at an isolated time and I looked forward to our chats.

### Knowledge

Some parents described their own and others’ knowledge, or lack of knowledge, as both challenging and isolating. Many commented that professionals in both secondary and primary care did not have sufficient information about CHD or their child’s specific heart condition:

There has been no one in the community or local hospital that had the answers.

And that they often knew more about their child than the local health professionals:

I found that we know lots more than they [community health professionals] did about her condition, which was both understandable...and terrifying in equal measures.

Parents also described their own stress associated with knowledge, in terms of either feeling that they did not know enough about their child’s condition or, conversely, the responsibility and burden of needing to communicate knowledge to local health professionals:

[I am]...just sick of explaining to everyone. I wish someone could tell me about him not the other way around.

In contrast, others talked about the efforts made by health professionals in the community to become more knowledgeable about CHD or to get advice from other professionals. Some parents described the information they were given by the specialist center, highlighting the degree to which tertiary centers varied in the information they gave families about local services and support networks, both in terms of what they provided and how they provided it.

### Psychological and Emotional Impact

Caring for a child after cardiac surgery can have a significant psychological and emotional impact, and parents described their anxiety, which, for some, developed into symptoms of post-traumatic stress or served to heighten their feelings of isolation. One parent talked about feeling unprepared for how life had changed and the tension between feeling grateful that her child had come through the surgery and was back home and not feeling able to tell the team how anxious she felt:

Your whole life changes and no one tells you that…I think I had a bit of posttraumatic stress but I was so grateful my baby was home I didn’t want to say how terrified I was constantly and how much I relived every moment.

It was evident that some parents felt disorientated and unsettled on returning home, with a number of parents describing that they felt in a “fog” when they got home after their baby’s surgery:

I was having panic attacks and quite “fog-like” for months, felt quite isolated but there seemed to be nobody really asking about the parents.

Many parents shared how lonely and scared they felt during those early weeks:

Felt out of my depth and very scared.

Some had clearly been traumatized by their experiences, and described feeling unsupported:

The child is discharged and the parents are left walking around in an often traumatised state with no suitable support.

And unable to share their experiences:

It has been the most traumatic year of my life and yet I feel I can’t really talk to anyone else about it.

Some parents also talked about their difficulty in perceiving themselves as a parent rather than just as someone providing medical care for their child because of the demands of the treatment regimen:

For a long time I struggled to feel like his mummy and not a nurse because that was all I seemed to be doing...medications, meds and more meds.

### Challenges

Parents provided insight into a number of challenges that they faced once home after their child’s surgery, which contributed to and compounded the overall feeling of isolation. Some of these challenges were related to practical issues, such as information, with parents describing a "roller-coaster of information and procedures" and how they received a lot of information in the hospital but nothing about being at home after discharge:

We got bombarded with info in hospital but once home there’s no information as to what to look out for.

Other parents described a number of practical difficulties associated with aspects of the treatment regimen, such as getting prescriptions:

At one point we were left without heart meds for one week. Doctor refused to prescribe...They told me they would phone the cardiologist to check. They didn’t. Needless to say my baby was getting sicker and sicker. I ended up crying down the phone pleading for a prescription.

Parents conveyed a sense of these difficulties undermining local health services and reducing parents’ trust and confidence in them.

Feeding was identified as a significant challenge by a number of the parents, which was related to the difficulties the baby had in feeding:

I had...this baby that struggled to feed, cried if I touched her and lost weight constantly. All I got was “just persevere and top her up with her NG.”

Challenges were also related to the time feeding took place and some of the practicalities for those children who were tube fed:

The first few days were a nightmare...I ended up syringing milk into her mouth as she wouldn’t take a bottle at all.

Some mothers also talked about breastfeeding, for example, one mother described feeling “lost and helpless” as she tried to feed her baby “without hurting her,” whereas others discussed the lack of support they experienced around feeding issues:

We had a horrendous 6 months and it was me that sought out support [for feeding issues] it wasn't just there for us.

Taking home a vulnerable baby after surgery was a further source of stress for a number of parents, related to the risk of complications, infections, or the fear of something going wrong, and parents conveyed a sense of waiting and watching for something to happen “…we feared an emergency could happen at any time” using words such as “horrendous,” “scared to death,” and “lost.” Several parents also identified that a lack of training had contributed to their anxiety:

We weren't offered anything. I researched myself and paid to go on a babies/children first aid course. Think it would've been massively useful (if only to instil some confidence in us as parents that we could cope if a situation had arisen) to have had some basic training or advice.

Finally, parents described the challenge of dealing with the reactions of others, particularly professionals, to them and their baby. For example, one parent talked about how she felt that her GP thought she was an “over-reactive mum,” whereas another parent felt that it was her baby whom her GP had issues with:

I found my doctor very “stand-offish” as if scared of him [baby].

### Mitigating Factors

Although some parents described the challenges of caring for a baby with a heart condition after cardiac surgery, many of them also talked about the things that helped and those factors which lessened the feelings of isolation. A number of the mitigating factors were related to the same topics which were challenges for other parents. For example, in a number of cases parents were provided with, or had access to, training and information before they left the specialist center:

We were asked in the hospital to give her meds under the nurse’s supervision so she could check we were administering them correctly. We were given all doses and medicines written down and plenty of syringes to take home. We were given a lot of info on care of her wound, what to do if she went blue etc and numbers for the CLN [cardiac liaison nurse], the ward and were told any queries just to call the ward direct which we did on a couple of occasions and got great and prompt advice.

Several parents were also given specific information about signs and symptoms to look for in their baby:

...told to look for blue lips. Fingers. If she got breathless tired sweaty while feeding also if oxygen levels go low.

A number of parents had open-access arrangements with their local hospital, which were clearly highly valued and helped to reassure parents:

We have open access at local hospitals and have [been] made to feel very welcome and nothing ever too big or small to come and see them.

Others described the accessibility of advice and the importance of that to them:

She was always on hand via phone or in person to answer questions and help explain stuff to us in layman’s terms. I honestly believe she made a world of difference to our ability to cope.

Although some parents had experienced negative reactions from health professionals, others had a very positive experience of care and support after hospital discharge from professionals in primary care, for example:

No problem was too small for the GP, she would phone the hospital if she needed to while I was waiting and send me straight away if she was worried about her.

A number of parents also described how their experience of secondary care had been excellent:

Our community nurse was brilliant, she even gave me her home number just in case I needed her. The health visitor was great, ENT [ear, nose and throat] feeding specialist was really helpful and still helps if I need her.

In some cases, babies were admitted to their local hospital in a step-down arrangement after treatment at the tertiary center, which parents valued:

...our local hospital would never let us go straight home after discharge from [specialist center], they would have [baby] in for 1-2 nights so THEY knew what to expect and how to treat, they were nervous of his heart but did a great job in ensuring they knew everything about him.

Parents also valued ongoing support from the cardiac liaison nurses in the specialist centers. As one mother said:

She [cardiac liaison nurse] was a familiar face in a whirlwind of unknowns...a friend in the know.

Support from people other than health professionals was also identified as an important factor for reducing isolation and facilitating coping. Parents described 3 main sources of nonmedical support as being other “heart families,” “online and charity support,” and the “support of family and friends.” One mother talked about wishing that she had had support:

...contact with someone who had “been there done that.”

Another mother saw parents in similar situations to hers as her first port of call for support:

I will ask the other heart mums and dads first as they usually know what’s what.

Although it was also evident that parents had to be ready to speak to other parents and that this was not the right approach for everyone, as one mother explained:

I'm still not at a point where seeking out other parents yet as I don't think I could share our story.

Online and charity contact were described as important and helpful sources of support, primarily as a means of having contact with other parents:

My lifeline throughout the whole experience was the “Heartline” charity forum (online) I got a tremendous amount of support and got in contact with 2 mums (both with heart children) who have supported me through the whole process.

The support from family and friends was mentioned less frequently than other sources of support, but those parents who did describe it saw it as an important facilitator of coping with the experience: “…it is the emotional support of friends and family that pulls you through,” which was also attributed to helping parents find some normality outside of their child’s heart condition and care:

We used to go to our local to see friends just to try and get some normal life and conversation.

## Discussion

### Study Findings

As far as we are aware, using a charity online forum as a systematic means of eliciting views from parents about their experiences of having a child with a health condition has not been undertaken previously within a research project. Collection of data using this method enabled us to reach a large number of potential participants, including those difficult-to-reach families who may find it more difficult or not wish to participate in projects that use more common methods of data collection such as focus groups, interviews, or questionnaires. Specifically, those families who were more isolated were able to participate, and this method offered a means of contributing to the project for those who were unable to participate in other strands of it. Furthermore, the ability to see other participants’ posts may have had a positive impact on the reluctant responder and encouraged and empowered them to engage.

The 2 aims of this element of the project were to elicit parents’ experiences of caring for a child with CHD and to collect information to inform the interviews with parents of high-risk infants. Both of these aims were achieved and, importantly, provided us with different information than that collected from other elements of the study. The population who participated in the online forum was broader than just parents of high-risk infants—rather, participants’ responses were reflective of a wider spectrum of both diagnoses and age of the child—increasing the likely generalizability of our results to the wider population of parents of children with CHD.

In terms of eliciting parents’ experiences of caring for a child with CHD, the main theme emerging from the forum was one of isolation, which parents described in terms of social and physical isolation and isolation related to knowledge. This finding was not as clearly articulated through the other methods of data collection in the wider project and provided unique insights not captured elsewhere. Physical and social isolation are common themes expressed by parents of children with health needs, including parents of children with other chronic illnesses [26], autism [27], and mothers of extremely preterm babies [28]. Parents also talked about the stress associated with knowledge about their child’s condition, in terms of feeling that they did not have enough knowledge, particularly in the early stages after hospital discharge, but also the burden of responsibility as the “keeper” of the knowledge about their child’s condition and the need to inform less knowledgeable professionals in primary care in particular. Over time, parents became “expert parents” in relation to the specifics of their child’s condition and treatment, a phenomenon seen in parents of children with other chronic conditions [29,30], but parents also expressed some ambivalence about this role associated with the need to assume responsibility for informing health professionals outside the tertiary center about their child’s condition.

The psychological and emotional impact of CHD on parents is well documented [31-34], and parents’ descriptions of their anxiety and symptoms of post-traumatic stress on the online forum corroborate findings in the literature [35,36]. What we were able to additionally elicit, however, was parents’ views about the specific challenges they faced following discharge after their child’s surgery and their perceptions about what helped mitigate their feelings of isolation and psychological distress. Key themes that emerged were related to information, training, practical issues, and support, and examples for each of these themes were provided of things which went well and things which went badly. Our recommendations from this and other elements of the study have been reported elsewhere [7-9].

Our third aim was to comment on the usefulness of the online forum. We were confident at the outset that there was a fit with the method and the research purpose. We chose the online forum because we needed a national approach: to explore with a range of parents from different parts of the country what it was like taking a baby home after congenital heart surgery. The lack of geographical boundaries was an advantage, as without this approach, the researchers would have needed to make long-distance trips to generate the qualitative data. Reduction in costs was a further advantage, in terms of travel costs and labor; generation of an automatic transcript was initially considered a further asset. On reflection, 3 challenges emerged. The first challenge was the natural selection of specific participants, resulting in our not reaching parents from ethnically diverse populations, discussed further as a limitation in our study. The second challenge was the theoretical saturation, limited when using asynchronous interaction, where exchanges take place over time, not in real time, and this limited the opportunities for the researcher to continue to sample and code until new instances of variation for existing themes have ceased to emerge. The third challenge was the creation of an automatic transcript, which meant that some important aspects of qualitative research methodology were missing from our study, such as the nonverbal cues reported in verbatim transcripts. Despite these challenges, creating commitment online was achieved. Participants remained engaged with the forum, and although some participants only responded to 1 or 2 questions, a group of approximately 30 parents responded to questions throughout (although not necessarily every question).

### Limitations

There were a number of limitations with this element of the study, which related to the method of collecting the data. The sample was predominantly white British respondents, most of whom were mothers, corroborating research which has identified that there are more female than male users of social media—particularly Facebook—and participants in online interactions tend to be predominantly white, younger, and highly educated [17,37]. One of the drivers for including this method of data collection was to enable parents who might otherwise not participate in research to have an opportunity to share their experiences. In particular, we were hoping that fathers and parents from ethnically diverse populations and those living in geographically difficult-to-reach areas would participate. Although parents came from a wide geographical area (all over the United Kingdom), we did not achieve our aim of capturing the views of fathers or parents whose ethnicity was not white British. To participate, parents required access to the CHF website and familiarity with Facebook, which meant that parents who did not speak English and were not able or willing to use social media could not participate. Furthermore, participants required a certain level of computer skills because of the requirement to register and login to the site. It is probable that parents from a more ethnically diverse population face challenges and have different experiences, which this method of data collection prevented us from capturing. In particular, participants who have received poorer education and do not have English as their native language are more likely to find it challenging to access hospital or community support, they receive less information, and find it more difficult to understand the information given, all of which may have a negative impact on their experience of caring for their child after hospital discharge. The selection bias in our study may therefore mean that our findings are an under-representation of the difficulties that these parents face when they take their baby home after heart surgery. Furthermore, families who are in contact with charities offering support may be more likely to engage with this type of research, thus limiting the representativeness of the sample. Finally, we did not have any “entry criteria” to the forum, other than the requirement to provide some demographic details, which did not include information about the child’s diagnosis. Although questions posted on the forum were related to discharge after infant cardiac surgery, some of the parents provided information and views about other stages of their journey (eg, after surgery in later childhood). Although we did not include these posts in our analysis where this was made explicit, it is possible that some posts were included where the time that parents were referring to was not identified as being other than following cardiac surgery in infancy.

We also had some challenges during the facilitation of the online forum, which was related to CHF staff leaving the organization during the time that the forum was running, which in turn meant that fewer probing questions were asked than intended. It was important that a neutral organization facilitated the forum rather than anyone from one of the specialist pediatric centers, particularly as at the time there was a national review taking place about the provision of pediatric cardiac services in the United Kingdom and whether services should be rationalized. It was also necessary that the forum was facilitated by an organization that had country-wide coverage and access to our specific target group (parents of children with CHD). CHF was clearly well placed to meet the requirements, and their staff turnover at a critical time for the project was unavoidable.

Online forums also have some inherent limitations compared with more traditional face-to-face methods of data collection. For example, nonverbal and contextual cues cannot be picked up on and specific participant comments cannot be probed, resulting in the potential loss of some richness of the data. Moreover, responses were generally much shorter than would be elicited in an interview. The automatically generated transcript, although being a benefit of this research method, was also not perfect, as has been identified previously in online forum research [38]. We did not collect information about the time spent on the site or number of visits participants made while the forum was running, and we also did not know if any participants had technical problems accessing the site at any time or whether potential participants failed to join the forum at all. However, it is also important to consider the aims of this part of the study and the fact that we were not conducting a qualitative study requiring data saturation but wanted to elicit information about parental experiences to inform the development of a topic guide for the in-depth interview in the next phase of the study.

Despite the limitations identified above, the online forum was a valuable component in our mixed-methods project. We have subsequently run another online forum for a different project, following a similar approach, in which we were able to address some of the challenges identified in this study related to the facilitation of the forum. However, reaching fathers and ethnically diverse families requires a different approach. We are aware of 1 support group in the United States that has designated part of its website specifically for fathers of children with complex heart disease, and 1 option would be to run a forum with this group, but we are not aware of anything similar for ethnically diverse families. Further work needs to be undertaken to identify more appropriate ways of engaging ethnic minorities in research, with specific attention given to the issues of culture and language.

### Conclusions

Use of an online forum provided a means of eliciting the experiences of a large number of parents in caring for their child after discharge from hospital following cardiac surgery. Parents engaged with the forum and were able to articulate what went well and what went less well, as well as sharing their stories and supporting each other through doing so. At an individual level, some parents clearly found participating in the forum a positive experience in itself, as one parent said:

Thank you for this forum, it has helped reading other people’s experiences and knowing I’m not alone.

Although this was not one of the specific aims of the project, it demonstrates the potential of social media as a mechanism for providing support and reducing isolation. Information gained from the forum was used to shape the questions for the parent interviews in a subsequent phase of the study, particularly in terms of the barriers and facilitators parents experienced for accessing care after discharge. Furthermore, the themes identified in the online forum have contributed—in conjunction with other findings from the study—to identifying ways of improving the provision of care and support for parents of high-risk babies following discharge after cardiac surgery. Both the methods and results of the study have, we believe, wider generalizability and offer the potential of shared learning for other populations of parents with children with lifelong health conditions, particularly those with complex conditions who are receiving care in multiple settings, as well as the health professionals delivering that care. CHD can be an isolating experience for parents, particularly when their children are infants or have been discharged after surgery, and this has implications for the care and support of these children and families in primary, secondary, and tertiary care.

## Abbreviations

CHD: congenital heart disease
CHF: Children’s Heart Federation
HV: health visitor
